# IMPDH2 suppression impedes cell proliferation by instigating cell cycle arrest and stimulates apoptosis in pediatric hepatoblastoma

**DOI:** 10.1007/s00432-024-05858-4

**Published:** 2024-08-01

**Authors:** Linman Li, Yichi Wu, Hong-ting Huang, June-kong Yong, Zicheng Lv, Yi Zhou, Xuelin Xiang, Jie Zhao, Zhifeng Xi, Hao Feng, Qiang Xia

**Affiliations:** 1 Department of Liver Surgery, Renji Hospital (Punan Branch), School of Medicine, Shanghai Jiao Tong University, Shanghai, 200127 China; 2 Clinical Research Unit, Renji Hospital, School of Medicine, Shanghai Jiao Tong University, Shanghai, 200127 China; 3 Shanghai Engineering Research Centre of Transplantation and Immunology, Shanghai, 200127 China

**Keywords:** Liver transplantation, Hepatocellular carcinoma, Neoadjuvant therapy, Recurrence-free survival, Immunotherapy, Hepatoblastoma, IMPDH2, Cell cycle, Pediatric cancer, Survival

## Abstract

**Background:**

Hepatoblastoma (HB) is the most common pediatric liver tumor, presenting significant therapeutic challenges due to its high rates of recurrence and metastasis. While Inosine Monophosphate Dehydrogenase 2(IMPDH2) has been associated with cancer progression, its specific role and clinical implications in HB have not been fully elucidated.

**Methods:**

This study utilized Quantitative Real-Time Polymerase Chain Reaction (qRT-PCR) and Tissue Microarray (TMA) for validation. Following this, IMPDH2 was suppressed, and a series of in vitro assays were conducted. Flow cytometry was employed to assess apoptosis and cell cycle arrest. Additionally, the study explored the synergistic therapeutic effects of mycophenolate mofetil (MMF) and doxorubicin (DOX) on HB cell lines.

**Results:**

The study identified a marked overexpression of IMPDH2 in HB tissues, which was strongly correlated with reduced Overall Survival (OS) and Event-Free Survival (EFS). IMPDH2 upregulation was also found to be associated with key clinical-pathological features, including pre-chemotherapy alpha-fetoprotein (AFP) levels, presence of preoperative metastasis, and the pre-treatment extent of tumor (PRETEXT) staging system. Knockdown of IMPDH2 significantly inhibited HB cell proliferation and tumorigenicity, inducing cell cycle arrest at the G0/G1 phase. Notably, the combination of MMF, identified as a specific IMPDH2 inhibitor, with DOX, substantially enhanced the therapeutic response.

**Conclusion:**

The overexpression of IMPDH2 was closely linked to adverse outcomes in HB patients and appeared to accelerate cell cycle progression. These findings suggest that IMPDH2 may serve as a valuable prognostic indicator and a potential therapeutic target for HB.

**Impact:**

The present study unveiled a significant overexpression of inosine monophosphate dehydrogenase 2 (IMPDH2) in hepatoblastoma (HB) tissues, particularly in association with metastasis and recurrence of the disease. The pronounced upregulation of IMPDH2 was found to be intimately correlated with adverse outcomes in HB patients. This overexpression appears to accelerate the progression of the cell cycle, suggesting that IMPDH2 may serve as a promising candidate for both a prognostic marker and a therapeutic target in the context of HB.

**Supplementary Information:**

The online version contains supplementary material available at 10.1007/s00432-024-05858-4.

## Introduction

Hepatoblastoma (HB) is the predominant pediatric liver cancer, typically affecting children aged below three years (Czauderna et al. 2014). Despite its rarity, the prevalence of HB has climbed annually in recent decades, constituting approximately 80% of pediatric liver malignancies (Bell et al. 2017; Linabery and Ross 2008). Clinical presentations often include abdominal distension and the detection of an abdominal mass through imaging. Elevated levels of AFP, a fetal hepatocyte-derived protein, are commonly observed and considered as a crucial tumor indicator for monitoring disease progression and response to comprehensive treatment. The rising incidence is speculated to be linked to improved survival rates among prematurely born or with extreme low weight, a population susceptible to HB (Heck et al. 2013; Fine Licht et al. 2012).

Primary therapeutic approaches for HB involve complete surgical resection and chemotherapy, with cisplatin-based regimens proving effective for unresectable tumors and contributing to increased survival rates (Davies et al. 2004). Advances in surgical techniques and postoperative chemotherapy have enhanced overall outcomes, yielding 5-year survival rates averaging 82% (Tulla et al. 2015). However, approximately 30% of patients, even with surgery and neoadjuvant chemotherapy, exhibit suboptimal outcomes (Aronson et al. 2019; Meyers et al. 2017). In cases of advanced HB, liver transplantation stands as the sole effective treatment option (Trobaugh-Lotrario et al. 2016).

Mechanistically, ever-increasing evidence implicates that HB originates from less differentiated cells, with disorders of developmental and self-regeneration pathways playing a pivotal role in hepato-carcinogenesis. The Wnt/beta-catenin pathway, in relation to stem cells, is critically important, influencing the activation and expansion of these cells during embryonic development and liver regeneration, thus fostering liver homeostasis (Monga 2015; Russell and Monga 2018). HB often exhibits resistance to chemotherapy at high levels (Marin et al. 2018). Unfortunately, the mechanisms underlying this resistance remain poorly understood.

IMPDH is recognized as a pivotal rate-controlling enzyme within the purine de novo synthesis pathway (Shu and Nair 2008). This enzyme comprises two isotypes: IMPDH1 and IMPDH2 (Natsumeda et al. 1990). with IMPDH1 exhibiting relatively stable expression in both normal and tumor cells. In contrast, IMPDH2 manifests significantly heightened expression levels across various malignant tumors (Collart et al. 1992). Accumulating evidence from previous investigations has underscored the substantial elevation of IMPDH2 expression, which correlates closely with tumor progression and a poor prognosis in a diverse array of malignancies (Zhou et al. 2014). Notably, overexpression of IMPDH2 has been identified in specific patient subgroups that demonstrate a diminished response to chemotherapy, thereby establishing it as an independent prognostic indicator for chemotherapeutic response and event-free survival (EFS) (Fellenberg et al. 2007). Recent research has unveiled markedly upregulated IMPDH2 expression in prostate, kidney, bladder, and liver cancers, suggesting its potential utility as a biomarker for these diseases(Yang et al. 2014; Zou et al. 2015; Jia et al. 2022). However, the precise role of IMPDH2 in the molecular mechanisms underlying hepatoblastoma (HB) remains insufficiently characterized within the current literature.

Cyclin-Dependent Kinase Inhibitor 1 A (CDKN1A), commonly referred to as p21, is a critical regulator of cell cycle progression, particularly during the G1 phase (Xiong et al. 1993). Functionally, p21 plays a multifaceted role in orchestrating cell cycle arrest, facilitating DNA repair, and regulating apoptosis (Mauro et al. 2012; Gartel and Tyner 2002). By inhibiting the activity of cyclin-dependent kinases (CDKs), p21 effectively prevents the transition of cells from the G1 to the S phase, thereby significantly contributing to the regulation of cell division and preventing aberrant cell proliferation. Furthermore, in response to DNA damage, p21 can induce cell cycle arrest, providing sufficient time for DNA repair to occur. Under certain conditions, p21 also participates in the modulation of programmed cell death, or apoptosis.

Mycophenolate mofetil (MMF), approved by the FDA in 1995 for the prevention of transplant graft failure (Lipsky 1996), is a widely utilized immunosuppressive agent in the post-organ transplantation setting. Acting as a prodrug, MMF is rapidly metabolized into mycophenolic acid (MPA), which targets IMPDH, thereby reducing the synthesis of guanine nucleotides (Ransom 1995; Suthanthiran and Strom 1997). In the therapeutic management of hepatoblastoma (HB), particularly in high-risk patients, a combination regimen of cisplatin and doxorubicin (DOX) is frequently employed (Trobaugh-Lotrario and Katzenstein 2012). However, both of these chemotherapy agents are associated with specific side effects. Cisplatin, for example, is known to induce severe ototoxicity. Dox, an anthracycline, exerts its therapeutic effects through a variety of mechanisms, including DNA intercalation, interference with type II topoisomerases, generation of free radicals, and inhibition of DNA and RNA synthesis (Zsíros et al. 2010). Cardiomyopathy stands as a recognized side effect of Dox therapy (Zsiros et al. 2013). Unfortunately, specific medications targeting DOX-induced cardiotoxicity are currently unavailable.

In the present study, our analysis of public databases revealed a significant overexpression of IMPDH2 in hepatoblastoma (HB) tissues, particularly in cases associated with metastasis and recurrence of the disease. Subsequently, we conducted an immunohistochemical assessment of IMPDH2 protein expression using tissue microarray (TMA) analysis. This was followed by an investigation into the correlations between IMPDH2 protein expression levels and various clinicopathological variables, as well as the overall survival (OS) and event-free survival (EFS) of the patients. Furthermore, we experimentally knocked down IMPDH2 in HB cell lines to determine its effects on cellular functions and its relationship with the cell cycle. Ultimately, our research led to the discovery of a synergistic interaction between mycophenolate mofetil (MMF), an inhibitor of the IMPDH2 target, and the chemotherapeutic agent doxorubicin (DOX).

## Materials and methods

### Patients and specimens

Tissue samples were obtained from a cohort of 129 patients diagnosed with hepatoblastoma (HB) at Renji Hospital, Shanghai, over a decade-long period from May 2013 to August 2023. For the study, we constructed tissue microarrays (TMAs) that included a total of 129 paraffin-embedded HB tumor specimens, 21 paraffin-embedded specimens of adjacent normal liver tissue from HB patients, and 10 paraffin-embedded specimens of normal liver tissue from controls. Furthermore, we randomly selected eight pairs of tumor and corresponding normal tissues for analysis via reverse transcription quantitative polymerase chain reaction (RT-qPCR). Surgical interventions for all patients included in the study were performed at Renji Hospital. The study protocol was approved by the Ethics Committee of Renji Hospital, and we obtained written informed consent from the guardians of all participating patients.

### Cell culture

The HB cell lines (Huh6, HepG2) and embryonic kidney cell lines (HEK293) were nurtured in Dulbecco’s modified Eagle’s medium, complemented with 10% fetal bovine serum. Incubation was sustained in a humidified environment at 37 °C, with 5% carbon dioxide. Cell passages were restricted to < 6 months before experimentation.

### Dataset acquisition and process

Three microarray datasets (GSE151347, GSE131329, GSE133039) sourced from the GEO database were utilized for investigating IMPDH2 expression in HB cancer tissues and normal tissues. Differential expression profiles of genes were generated through GEO2R analysis. Subsequent analysis and visualization of Gene Ontology (GO) results were conducted employing R Studio software (version 4.2.3). The HSA Synergy Score was assessed utilizing the online tool SynergyFinder. (https://tangsoftwarelab.shinyapps.io/synergyfinder/_w_f98c38d7/#!/dashboard ).

### TMA staining and immunohistochemistry (IHC) assay

TMA sections, each 4 μm thick and prepared using a Leica RM2245 microtome, underwent heat treatment at 60 °C for 30 min, followed by three cycles of immersion in xylene for 10 min per cycle and subsequent immersion in absolute ethanol for 10 min per cycle. After rinsing with running water, experiments were conducted. IHC was performed to investigate IMPDH2 expression in the TMA, utilizing anti-IMPDH2 as the primary antibody. A semi-quantitative scoring system was devised, considering both the proportion of cells displaying positive staining and the intensity of staining. The positive staining proportions were graded as follows: 0 for absence, 1 for 0–25%, 2 for 25–50%, 3 for 50–75%, and 4 for 75–100%. Staining intensity was rated on a scale of 0 for absence, 1 for weak, 2 for moderate, and 3 for strong. The combined score was calculated by multiplying these two sub-scores, allowing for the classification of samples into categories reflecting negative expression (0), low expression (1–3), moderate expression (4–6), and high expression (9–12). Two blinded pathologists independently assessed the IHC staining results without access to clinicopathologic data.

### Western blot

Twenty micrograms of protein samples were applied onto 10% sodium dodecyl sulfate-polyacrylamide gel electrophoresis. After separation, these samples were transferred from the gel to nitrocellulose membranes (Millipore, MA, USA), which underwent blocking using 5% bovine serum albumin for 1 h. Subsequently, the membranes were subjected to an overnight incubation at 4 °C with anti-β-actin, IMPDH2, or specific antibodies as indicated. Following washing with PBST, secondary antibody incubation was conducted for 1 h, and subsequent visualization was achieved by exposure of the membranes to photographic film. Table S2 contains detailed information about the utilized antibodies.

### Quantitative real-time PCR (RT-qPCR)

Real-time PCR analyses were conducted in triplicate, utilizing 25 ng of cDNA per reaction and 10 µM forward and reverse primers, employing 2× ChamQ SYBR Color qPCR Master Mix (Vazyme Biotech) on a Bio-Rad CFX 96 system (Bio-Rad, California, USA). Details of primer sequences are available in Tables S3. The PCR cycling protocol initiated with a 30-second denaturation at 95 °C, succeeded by 40 cycles consisting of 10 s at 95 °C for denaturation and 30 s for annealing/elongation. Normalization of target gene expression to GAPDH was performed, and the relative expression of target genes was determined employing the 2^−ΔΔCT^ method.

### Cell proliferation and colony formation assays

Cell proliferation was evaluated utilizing the CCK-8 Kit (Beyotime, China). Assessment of cell colony-forming ability post-IMPDH2 silencing was conducted via colony formation assay. Cells, transfected with shIMPDH2 or shNC controls, were seeded into 12-well plates and incubated for a duration of 2 weeks. Subsequently, they were rinsed twice with PBS, followed by fixation using 4% formaldehyde for 15 min, staining with crystal violet for an additional 15 min, and subsequent photographic documentation.

### Transwell

HepG2 and Huh6 cell lines were maintained in a serum-depleted medium within the upper transwell chamber (Corning, USA) containing an 8.0 μm pore polycarbonate membrane. The lower chamber was supplemented with medium containing 20% FBS. After a 24-hour incubation period, cells that traversed the membrane and adhered to its undersurface were fixed and stained using crystal violet.

### Cell transfection

Lentiviral vectors containing IMPDH2 shRNA plasmids (shIM#1, shIM#2, Supplement) and an empty vector (shNC) were procured from Shanghai Jiaotong University School of Medicine. To develop stable IMPDH2 knockdown HB cell lines, lentiviral particles were produced through co-transfection of the shIMPDH2 plasmid with a mix of packaging plasmids (pMDL, VSVG, pRSV-Rev at a ratio of 5:3:2) using PEI MAX transfection reagent (Polysciences, 24765-100) in HEK-293T cells. The viral particles were collected 48–72 h post-transfection. Subsequently, HepG2 and Huh6 cells were exposed to the lentivirus containing IMPDH2 shRNA or control lentivirus for 48 h and subsequently subjected to puromycin screening to select for cells exhibiting stable knockdown.

### Flow Cytometry

Cell cycle analysis was performed using the Cell Cycle Staining Kit, while apoptosis assessment utilized the Annexin V-FITC/PI apoptosis Kit (MULTI SCIENCES, Shanghai, China) as per the manufacturer’s protocols. BD FACSCanto II (USA) was employed for cell collection and analysis. ModFit LT 3.2 software was utilized to compute cell cycle distribution and apoptosis ratio, and Flowjo 10.8.1 was used for the visualization of apoptosis-related images.

### Tumorigenesis assays in nude mice

All animal experiments adhered to the Institutional Animal Care and Use Committee guidelines at Shanghai Jiaotong University. Four-week-old male BALB/c nude mice were randomly allocated to specified groups. Subcutaneous tumor models were established using HepG2 and Huh6 cells. The study comprised three groups: two experimental groups with IMPDH2 knockdown (HepG2 shIM#1, HepG2 shIM#2) and one control group (HepG2 shNC), each consisting of three nude mice. Each mouse received subcutaneous injections of HepG2 cells (5 × 10^6^ in 50% Matrigel™, BD Biosciences). The huh6 groups were similar to HepG2 groups, differing only in the number of injected cells (1.0 × 10^7^ in 25% Matrigel™). Tumor volume was assessed every 2 days using calipers and calculated using the formula (width^2^ x length)/2. Finally, humane euthanasia using carbon dioxide was performed, and tumors were harvested and prepared for further analysis.

### Statistical analysis

All experiments were conducted in triplicate, and GraphPad Prism software was utilized for data analysis. Results are expressed as mean ± standard error of mean (SEM). Unpaired and paired two-tailed student’s t-tests were employed as the analytical method to assess differences between the two groups. Kaplan–Meier methodology was applied for OS and EFS calculations, and between-group disparities were evaluated using the log-rank test. Clinicopathological characteristics in HB were subjected to analysis using χ2 tests conducted via SPSS software. A P value less than 0.05 was considered statistically significant (* *P* < 0.05, ** *P* < 0.01, *** *P* < 0.001).

## Results

### IMPDH2 is highly expressed in HB tissues and is associated with metastasis and recurrence

**Fig. 1:**
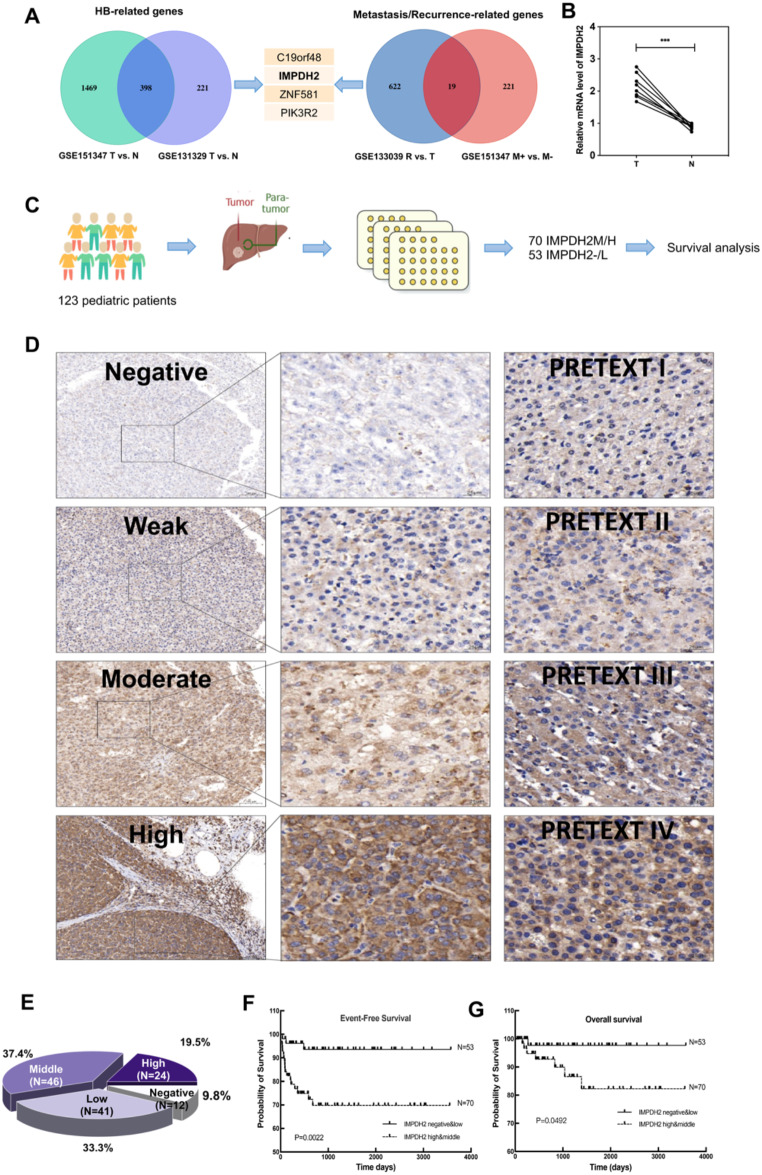
IMPDH2 is highly expressed in HB tissues and is associated with metastasis and recurrence. A. Left, Venn diagrams displayed DEGs retrieved from the GEO database (GSE151347 T vs. N, GSE131329 T vs. N); the Right, illustrated Venn diagrams of DEGs from the GEO database (GSE133039 R vs. T, GSE151347 M + vs. M-); The middle section depicted the collective genes overlapping across all datasets. *P* < 0.001, log2FC > 1. B. The RT-qPCR analysis unveiled the mRNA expression levels of IMPDH2 in HB tissues, encompassing eight pairs of tumors and their corresponding paired normal tissues. C: The schematic diagram illustrating the collection, classification, and analysis of tumor samples. D: Representative images and proportion of HB TMA with different staining intensities. E: The proportion of HB TMA with different staining intensities. F. Patient EFS between IMPDH2^−/low^ and IMPDH2^medium/high^ group. G. Patient OS between IMPDH2^−/low^ and IMPDH2^medium/high^ group

To explore the differential expression of IMPDH2 in hepatoblastoma (HB) relative to healthy tissue, an extensive analysis was undertaken, leveraging datasets from the Gene Expression Omnibus (GEO). The datasets included GSE151347, GSE131329 (comparing tumor to non-tumor tissue), GSE133039 (analyzing recurrence versus non-recurrence), and again GSE151347 (examining metastasis versus non-metastasis). Our results consistently revealed a significant overexpression of IMPDH2 in HB tissue, which was particularly pronounced in cases associated with metastasis and recurrence in HB patients **(**Fig. 1A**)**. To substantiate the validity of these public data, we conducted a preliminary assessment of IMPDH2 expression levels using reverse transcription quantitative polymerase chain reaction (RT-qPCR) in a series of HB tissues and their matched healthy liver tissue counterparts at the transcriptome level. These analyses confirmed a marked upregulation of IMPDH2 in HB patients **(**Fig. 1B**)**. With the goal of achieving a comprehensive understanding of IMPDH2 expression at the protein level, we constructed a tissue microarray (TMA) encompassing a considerable number of HB cases. Examination of the TMA with an IMPDH2 antibody led to the clear stratification of cases into negative, low, moderate, and high expression groups based on IMPDH2 levels **(**Fig. 1C-D**)**. It is noteworthy that the vast majority, 90.2%, showed positive IMPDH2 expression **(**Fig. 1E**)**. Furthermore, stark differences were observed between the high/medium and negative/low IMPDH2 expression groups with respect to pre-chemotherapy alpha-fetoprotein (AFP) levels, pre-operative metastasis, and the PRETEXT staging system **(**Table 1), along with a notable correlation with Ki67 expression. In addition, HB patients with negative/low IMPDH2 expression exhibited significantly better overall survival (OS) and event-free survival (EFS) compared to those with high/medium IMPDH2 expression **(**Fig. 1F-G**)**. In aggregate, these observations strongly suggest that the pronounced overexpression of IMPDH2 adversely affects the prognosis of HB patients. Moreover, IMPDH2 holds promise as a potential prognostic and risk prediction biomarker.

**Table 1:** Clinicopathologic characteristics of HB TMA

Characteristic	IMPDH2^−/L^ (*N* = 53)	IMPDH2^M/H^ (*N* = 70)	*P*
**Gender**			0.713
Male	27	38	
Female	26	32	
**Age(years)**	2.67 ± 2.73	3.30 ± 2.42	0.029
**Histologic subtypes**			0.613
Fetal or embryonal	29	42	
Mixed epithelial-mesenchymal	15	16	
Small cell undifferentiated (SCU)	0	1	
**Surgical Procedure**			0.414
LR	42	51	
LT	11	19	
**Ki67 levels**			0.075
0 ~ 10%	29	20	
10 ~ 30%	4	17	
30%~	14	18	
**Pre-chemotherapy AFP ng/mL**			**0.013**
~ 999	15	12	
1000 ~ 9999	8	13	
10,000~	11	32	
**Pre-operative metastasis**			**0.009**
Yes	3	16	
No	50	54	
**PRETEXT stages**			**0.011**
I	16	10	
II	20	29	
III	11	12	
IV	4	18	

### Knockdown of IMPDH2 suppresses HB cell malignancy in vitro

**Fig. 2:**
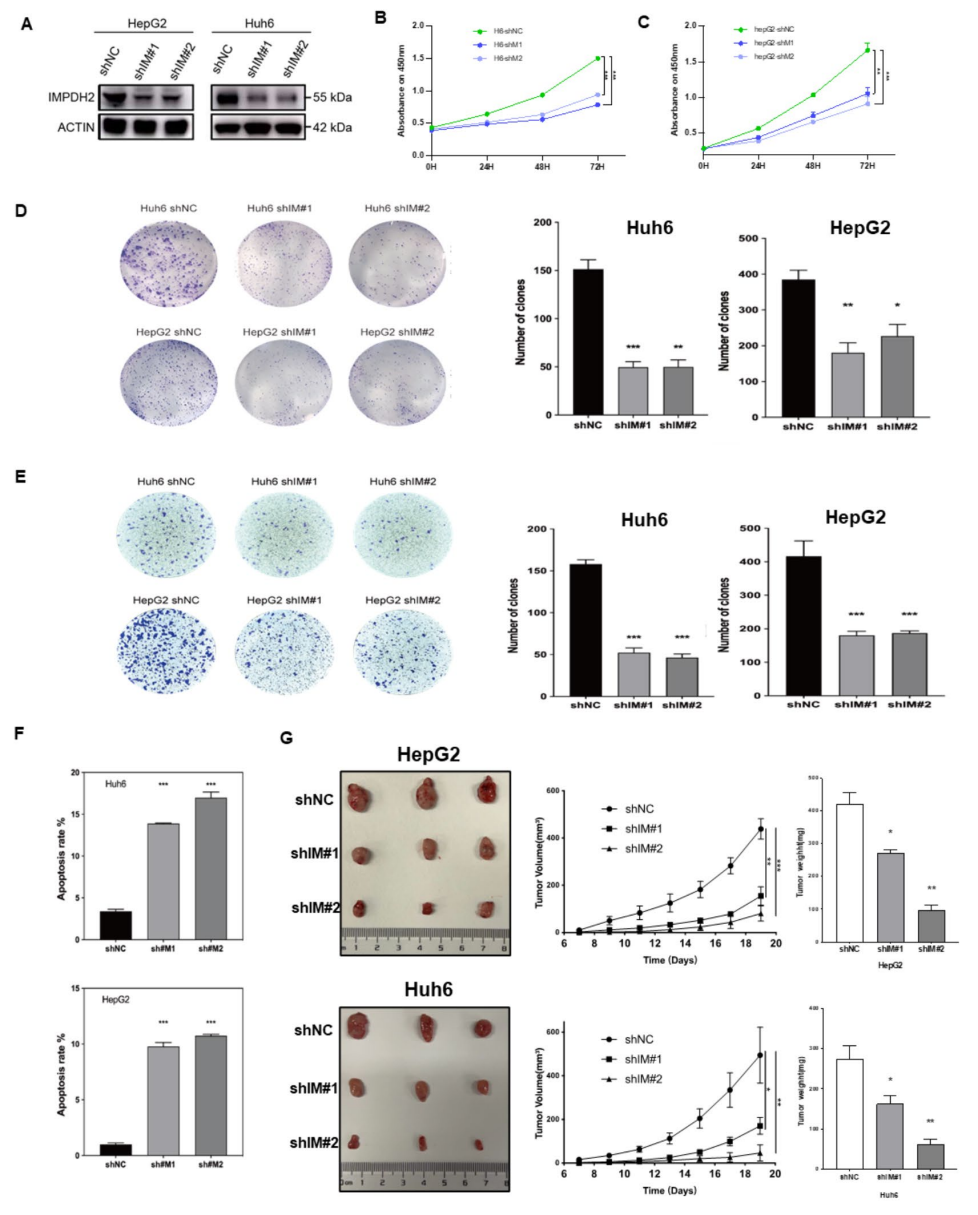
Knockdown of IMPDH2 suppresses HB cell malignancy in vitro and in vivo. A. Western blot assays validating the efficiencies of IMPDH2 knockdown in HB cell lines. B-C. The CCK-8 assay was employed to evaluate proliferative potential in HB cell lines post transfection with IMPDH2 shRNA (shIM#1 and shIM#2) and control shRNA. (*n* = 3, t-test). D. Colony formation assays were utilized to assess the proliferative capacity of HB cell lines following transfection with IMPDH2 shRNA and control shRNA. (*n* = 3, t-test). E. Transwell assays were employed to evaluate the metastatic potential of HB cell lines subsequent to transfection with IMPDH2 shRNA and control shRNA. (*n* = 3, t-test). F. A Flow cytometry analysis was used to assess the apoptosis rate of HB cell lines post transfection with IMPDH2 shRNA and control shRNA. (*n* = 3, t-test). G. The in vivo tumorigenic potential of HepG2, Huh6 cells transfected with IMPDH2 shRNA and control shRNA, respectively. B-C. Assessment of tumor volumes and weights of transfected HepG2 and Huh6 cells was conducted every 2 days, respectively

To elucidate the biological role of inosine monophosphate dehydrogenase 2 (IMPDH2) in hepatoblastoma (HB) cells, we experimentally downregulated IMPDH2 expression in HB cell lines **(**Fig. 2A**)**. Subsequently, the proliferation rate of these IMPDH2-knockdown cell lines was evaluated using the CCK-8 assay. Our results demonstrated a significant reduction in the proliferative capacity of the HB cell lines upon IMPDH2 suppression **(**Fig. 2B-C**)**. In parallel, the clonogenic potential of the HB cell lines was markedly diminished following IMPDH2 knockdown **(**Fig. 2D**)**. Furthermore, transwell migration assays indicated a pronounced inhibition of the migratory capacity of the HB cell lines post-IMPDH2 knockdown **(**Fig. 2E**)**. Importantly, previous studies have reported a significant increase in apoptosis following IMPDH2 inhibition in cell lines derived from triple-negative breast cancer and diffuse large B-cell lymphoma(Wang et al. 2021; Gao et al. 2023). Prompted by these findings, we investigated the impact of IMPDH2 knockdown on apoptosis using flow cytometry. Our analysis revealed a substantial increase in apoptotic cell death following IMPDH2 knockdown **(**Fig. 2F**)**. Collectively, these observations suggest that IMPDH2 plays a critical role in promoting the malignant progression of HB cells while concurrently suppressing apoptotic processes within these cell lines.

### Knockdown of IMPDH2 inhibits tumor proliferation in vivo

To further investigate the impact of IMPDH2 on hepatoblastoma (HB) proliferation in vivo, HepG2 and Huh6 cells were transfected with stable short hairpin negative control (shNC) and short hairpin IMPDH2 (shIMPDH2) constructs, specifically shIM#1 and shIM#2. These cells were then inoculated into nude mice. After a period of 19 days following the injection of the HepG2 cell line, xenograft tumors formed at the injection site in all mice. Notably, the group treated with shIMPDH2 demonstrated significantly reduced tumor growth rates when compared to the shNC group. A comparable trend was observed in the results from the Huh6 cell line **(**Fig. 2G**)**. These findings corroborate that the suppression of IMPDH2 markedly hinders tumor formation and growth.

### IMPDH2 regulates the progression of HB through the cell cycle signaling pathway

**Fig. 3:**
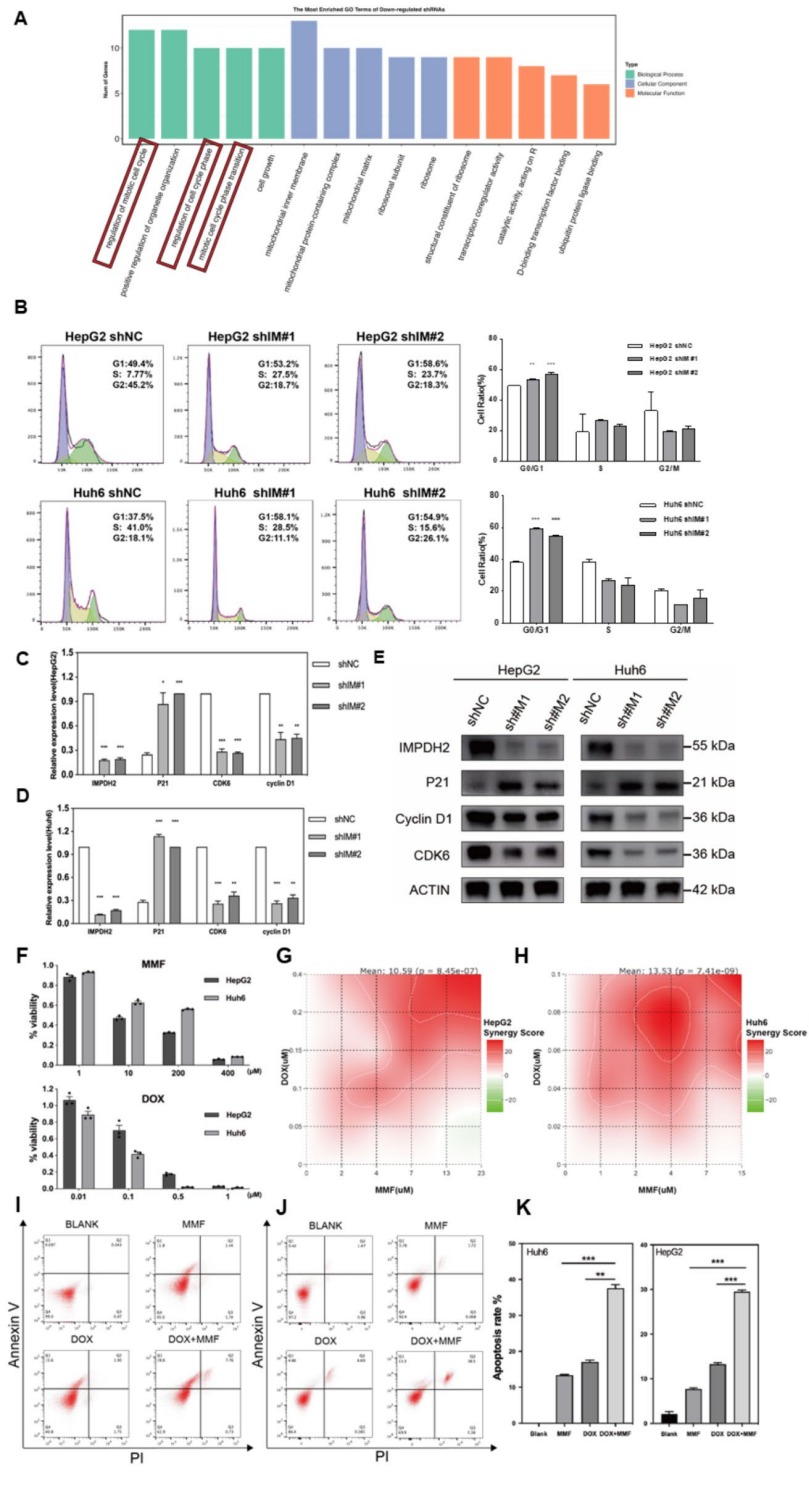
IMPDH2 regulates the progression of HB through the Cell Cycle Signaling Pathway. Combination of Doxorubicin with MMF remarkably reduced tumor proliferation and promoted apoptosis in vitro. A. Results of Gene Ontology analysis in HB cell lines. B. Analysis of cell cycle distribution in HB cell lines using flow cytometry following IMPDH2 knockdown: graphical representation of cell cycle distribution and statistical analysis. C-D. Quantification of IMPDH2, P21, CDK6, and cyclin D1 mRNA expression levels in HB cell lines transfected with IMPDH2 shRNA and control shRNA, assessed via real-time PCR. E. Validation of changes in cell cycle-related proteins following knockdown of IMPDH2 using Western blot assays. F. Cellular viability of HB cell lines post-administration of DOX and MMF was evaluated utilizing the CCK-8

To delineate the molecular mechanisms underlying the inhibitory effects observed upon IMPDH2 knockdown, we performed RNA sequencing on hepatoblastoma (HB) cell lines with IMPDH2 knockdown. The analysis of differentially expressed genes (DEGs) revealed a significant upregulation of genes linked to cell cycle regulation. Gene Ontology (GO) term enrichment analysis underscored the role of these genes in processes such as the regulation of the mitotic cell cycle, cell cycle phase control, and the transition through the mitotic cell cycle phase **(**Fig. 3A**).** This prompted us to focus on the influence of IMPDH2 on the cell cycle in HB cells. The impact of IMPDH2 knockdown on the cell cycle was evaluated using flow cytometry. We observed a pronounced accumulation of cells in the G0/G1 phase in the IMPDH2 knockdown group compared to the IMPDH2 short hairpin negative control (shNC) group, accompanied by a significant decrease in the G2/M phase cell population **(**Fig. 3B**)**. The increase in apoptosis observed following IMPDH2 knockdown **(**Fig. 2F**)** supports our inference that IMPDH2 knockdown results in G0/G1 cell cycle arrest, which subsequently initiates apoptosis. It is noteworthy that the cell cycle inhibitory protein p21 was significantly upregulated in the IMPDH2 knockdown group relative to the IMPDH2 shNC group. In contrast, genes associated with the G1/S transition, such as CDK6 and cyclin D1, showed marked downregulation. These results were further validated by reverse transcription quantitative polymerase chain reaction (RT-qPCR) analysis **(**Fig. 3C-D**)** and confirmed through Western blot analysis **(**Fig. 3E). In summary, our results elucidate that IMPDH2 knockdown induces G0/G1 phase cell cycle arrest and triggers apoptosis in HB cell lines. This is achieved through the upregulation of the cell cycle inhibitory protein p21 and the concurrent downregulation of cell cycle transition genes CDK6 and cyclin D1.

### Combination of doxorubicin with mmf remarkably reduced tumor proliferation and promoted apoptosis in vitro

Cisplatin, a chemotherapy agent extensively utilized in the treatment of hepatoblastoma (HB), exerts its therapeutic effect by disrupting DNA repair mechanisms, thereby inhibiting cancer cell growth. The efficacy of cisplatin is significantly enhanced when administered in combination with doxorubicin (DOX). Mycophenolate mofetil (MMF) functions as an inhibitor of purine synthesis by targeting inosine monophosphate dehydrogenase (IMPDH) activity, which in turn suppresses the de novo biosynthesis of purine nucleotides. Our hypothesis was centered on the potential synergistic effect of combining DOX’s inhibition of DNA synthesis with MMF’s blockade of de novo DNA synthesis, thereby amplifying their therapeutic impact on tumors. To evaluate this hypothesis, HB cell lines were treated with DOX, MMF, and their combination. The resulting dose-response relationships for each drug were depicted (Fig. 3F). Subsequently, the computation of the HSA Synergy Score revealed the striking superiority of the DOX-MMF combination treatment (40 nM DOX and 1 µM MMF) over the individual drugs (Fig. 3G-H). These results indicate that the addition of a specific dosage of MMF can significantly enhance the tumor-suppressive effects of DOX. Furthermore, our examination of the impact of combination therapy on cell apoptosis clearly demonstrated a pronounced pro-apoptotic effect of the combined treatment when compared to monotherapy (Fig. 5I-K). Consequently, we propose that optimizing the concentration of MMF in conjunction with DOX administration could lead to improved treatment outcomes. This strategy could potentially enable the use of reduced DOX dosages, thereby minimizing the side effects associated with MMF.

## Discussion

As the most prevalent class of primary liver malignancies, hepatoblastoma (HB) exhibits a pronounced propensity for metastasis, particularly to the lungs, and often displays inherent resistance to chemotherapy. The poor prognosis and overall survival rates observed in HB patients are largely attributable to recurrent episodes and the dissemination of metastases. Consequently, our study was designed to explore the fundamental mechanisms and potential biomarkers underlying HB.

Within this investigation, elevated IMPDH2 expression was observed among HB patients, which correlated with unfavorable prognostic indicators. Silencing IMPDH2 expression resulted in a significant deceleration of in vitro cellular proliferation and induced cell cycle arrest at the G0/G1 phase in HB cells. Additionally, IMPDH2 suppression led to an increase in P21 expression, disrupting the functionality and binding capacities of CDK6 and Cyclin D1, thereby promoting cellular apoptosis and inhibiting cellular proliferation. These observations position IMPDH2 as a significant contributor to HB progression and suggest its potential as a promising prognostic marker for this disease.

In our study, we collected postoperative samples from HB patients at Renji Hospital spanning the past decade. These samples encompassed tumor tissues as well as paired adjacent normal tissues, with comprehensive clinical data gathered from the corresponding patients. Utilizing these samples, we established the most extensive hepatoblastoma tissue microarray (TMA) to date. Subsequent analysis of publicly accessible databases concerning HB revealed a notable increase in IMPDH2 expression at the transcriptional level, significantly correlating with instances of metastasis and recurrence.

Further evaluation of IMPDH2 protein expression using the TMA platform confirmed its elevated levels in HB. Integration of clinical parameters revealed a significant association, with patients exhibiting heightened IMPDH2 expression tending to present at older ages (Table 1), validating prior research findings (Haeberle et al. 2020). Moreover, approximately 20% of pediatric HB cases present with metastatic disease at diagnosis, predominantly affecting the lungs and significantly impacting patient prognosis(Hu et al. 2021; Angelico et al. 2019). Our investigation identified a strong correlation between HB metastasis and increased IMPDH2 expression. Elevated AFP levels serve as a crucial diagnostic criterion for HB, with the majority of patients demonstrating abnormally high levels. Clinical conditions often parallel AFP levels, and existing literature suggests that both low and extremely high AFP levels in pediatric HB patients correspond to poorer prognoses (Meyers et al. 2017; Schweinitz et al. 1997). Our findings indicate that among patients with heightened IMPDH2 expression, there is a notably higher prevalence of individuals with AFP levels exceeding 10,000 ng/mL, aligning with previous literature findings in patients exhibiting high IMPDH2 expression. Remarkably, both overall survival (OS) and event-free survival (EFS) demonstrated a significant decline in the cohort characterized by increased IMPDH2 expression, mirroring outcomes observed in various solid malignancies(Duan et al. 2018; He et al. 2018; Shireman et al. 2021). These outcomes underscore the robust reliability of our TMA platform in identifying novel biomarkers. We propose that our platform holds promise for uncovering additional pertinent biomarkers in future research endeavors.

In addition to the cell line sequencing outcomes following IMPDH2 knockdown, which affirmed the correlation between IMPDH2 and the cell cycle, our analysis of GSE131329 yielded substantial findings. The Kyoto Encyclopedia of Genes and Genomes (KEGG) pathway examination (Supplemental Fig. 1) identified the cell cycle and Wnt signaling pathway as the foremost pathways of significance. The Wnt/beta-catenin pathway, a pivotal developmental pathway associated with progenitor/stem cells, plays a critical role in activating and expanding these cells during embryogenesis and liver regeneration, thereby facilitating hepatic homeostasis(Monga 2015; Russell and Monga 2018). Notably, HB demonstrates the highest incidence of beta-catenin mutations among human cancers, closely associated with aberrant Wnt/beta-catenin signaling, reaching rates of up to 90%. This not only validates the reliability of the dataset but also substantiates our investigation. This is evident in our observation that the inhibition of IMPDH2 expression in HB cells resulted in the arrest of the cell cycle at the G0/G1 phase.

Purine nucleotides hold a pivotal role in governing cell cycle dynamics, where IMPDH2 actively participates in their synthesis. These nucleotides are indispensable for DNA and RNA construction, fundamental processes driving cell growth and division. Consequently, IMPDH2 indirectly modulates cell cycle progression by influencing the synthesis of purine nucleotides. Our findings substantiate an expanding body of literature indicating IMPDH2 involvement across diverse cancer types. Notably, heightened IMPDH2 expression in colon cancer cells has been linked to methotrexate resistance(Peñuelas et al. 2005). In glioblastoma, IMPDH2 amplifies rRNA and tRNA synthesis, fostering accelerated cell proliferation(Kofuji et al. 2019). Furthermore, elevated IMPDH2 levels have demonstrated suppression of cancer cell apoptosis through the regulation of multifaceted pathways, including the PI3K/AKT/mTOR and PI3K/c-Myc/AFF4 pathways (Gao et al. 2023; Ni et al. 2023). In this current investigation, we elucidate a novel mechanism whereby IMPDH2 augments the proliferative and tumorigenic potential of HB cells by modulating the cell cycle pathway.

Surprisingly, our investigation unveiled mycophenolate mofetil (MMF), an FDA-approved medication widely used for post-transplant rejection, as a specific inhibitor of IMPDH2(Lipsky 1996). Its combination with doxorubicin (DOX) demonstrated a notably synergistic effect. Therefore, we postulate that, for post-transplant HB patients, utilizing MMF, as opposed to other immunosuppressants, may offer a dual benefit of anti-rejection and tumor suppression. This hypothesis warrants further substantiation through clinical investigations, forming the cornerstone of our subsequent research endeavors.

In summary, our identification of IMPDH2 as a novel prognostic marker and therapeutic target in HB, particularly in cases of metastasis or recurrence, suggests that targeting this factor holds potential to enhance patient prognosis and overall quality of life.

## Electronic supplementary material

Below is the link to the electronic supplementary material.

Supplementary Material 1

## Data Availability

No datasets were generated or analysed during the current study.
