# Supplementary material for: IMPDH2 suppression impedes cell proliferation by instigating cell cycle arrest and stimulates apoptosis in pediatric hepatoblastoma
Source: J Cancer Res Clin Oncol. 2024 Aug 1;150(8):377. doi: 10.1007/s00432-024-05858-4 (PMC11291533; doi:10.1007/s00432-024-05858-4)

**Supplement**

**IMPDH2 shRNA**

| GENE | Full Hairpin Sequence | Sense Sequence |
| --- | --- | --- |
| IMPDH2 | TGCTGTTGACAGTGAGCGACCGGACAGACCTGAAGAAGAATAGTGAAGCCACAGATGTATTCTTCTTCAGGTCTGTCCGGGTGCCTACTGCCTCGGA | CGGACAGACCTGAAGAAGA |
| IMPDH2 | TGCTGTTGACAGTGAGCGCCAAGTACATCAAAGACAAATATAGTGAAGCCACAGATGTATATTTGTCTTTGATGTACTTGATGCCTACTGCCTCGGA | AAGTACATCAAAGACAAAT |

|  | Antibody |
| --- | --- |
| IPMDH2 | CatNo.12948-1-AP, proteintech |
| P21 | p21 Waf1/Cip1 (12D1) Rabbit mAb #2947,CST |
| CDK6 | CDK6 (D4S8S) Rabbit mAb #13331,CST |
| cyclin D1 | Cyclin D1 (E3P5S) XP® Rabbit mAb #55506,CST |
| Actin | β-Actin Antibody #4967,CST |

|  | Forward | Reverse |
| --- | --- | --- |
| IPMDH2 | AGTGGCTCCATCTGCATTACGC | GGATTCCTCCATCAGCAATGACC |
| P21 | AGGTGGACCTGGAGACTCTCAG | TCCTCTTGGAGAAGATCAGCCG |
| CDK6 | GGATAAAGTTCCAGAGCCTGGAG | GCGATGCACTACTCGGTGTGAA |
| cyclin D1 | TCTACACCGACAACTCCATCCG | TCTGGCATTTTGGAGAGGAAGTG |
| GAPDH | GTCTCCTCTGACTTCAACAGCG | ACCACCCTGTTGCTGTAGCCAA |


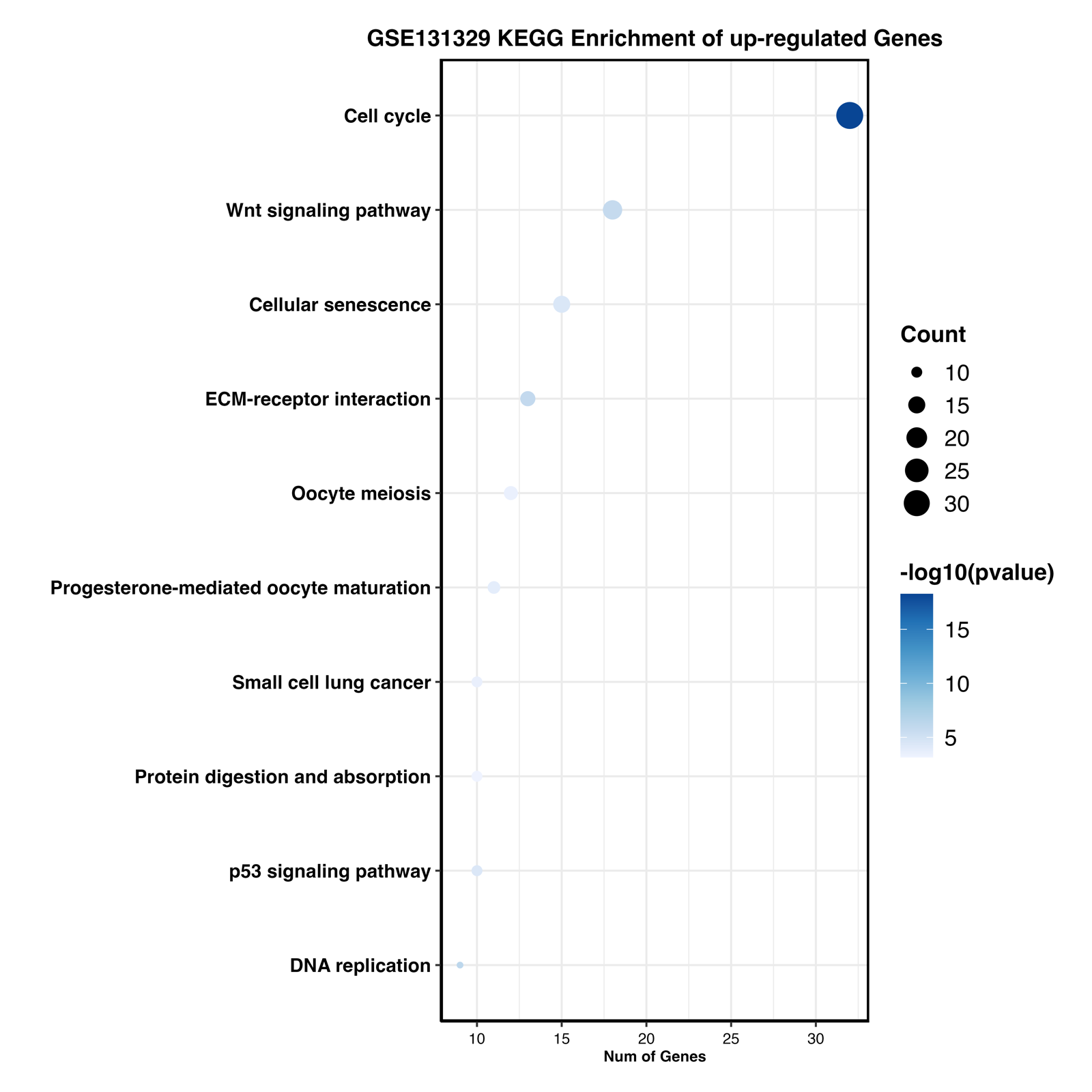

Supplement: Supplementary file 1 — Supplementary Material 1 [file 432_2024_5858_MOESM1_ESM.docx]
